# Stem Cells Propagate Their DNA by Random Segregation in the Flatworm *Macrostomum lignano*

**DOI:** 10.1371/journal.pone.0030227

**Published:** 2012-01-19

**Authors:** Freija Verdoodt, Maxime Willems, Stijn Mouton, Katrien De Mulder, Wim Bert, Wouter Houthoofd, Julian Smith, Peter Ladurner

**Affiliations:** 1 Nematology Unit, Department of Biology, Ghent University, Ghent, Belgium; 2 Laboratory of Pharmaceutical Technology, Department of Pharmaceutics, Ghent University, Ghent, Belgium; 3 Hubrecht Institute, Royal Netherlands Academy of Arts and Sciences and University Medical Center Utrecht, Utrecht, The Netherlands; 4 Department of Clinical Chemistry, Microbiology, and Immunology, Ghent University, Ghent, Belgium; 5 Department of Biology, Winthrop University, Rock Hill, South Carolina, United States of America; 6 Institute of Zoology and CMBI, University of Innsbruck, Innsbruck, Austria; National Institute on Aging, United States of America

## Abstract

Adult stem cells are proposed to have acquired special features to prevent an accumulation of DNA-replication errors. Two such mechanisms, frequently suggested to serve this goal are cellular quiescence, and non-random segregation of DNA strands during stem cell division, a theory designated as the immortal strand hypothesis. To date, it has been difficult to test the *in vivo* relevance of both mechanisms in stem cell systems. It has been shown that in the flatworm *Macrostomum lignano* pluripotent stem cells (neoblasts) are present in adult animals. We sought to address by which means *M. lignano* neoblasts protect themselves against the accumulation of genomic errors, by studying the exact mode of DNA-segregation during their division.

In this study, we demonstrated four lines of *in vivo* evidence in favor of cellular quiescence. Firstly, performing BrdU pulse-chase experiments, we localized ‘Label-Retaining Cells’ (LRCs). Secondly, EDU pulse-chase combined with Vasa labeling demonstrated the presence of neoblasts among the LRCs, while the majority of LRCs were differentiated cells.We showed that stem cells lose their label at a slow rate, indicating cellular quiescence. Thirdly, CldU/IdU− double labeling studies confirmed that label-retaining stem cells showed low proliferative activity. Finally, the use of the actin inhibitor, cytochalasin D, unequivocally demonstrated random segregation of DNA-strands in LRCs.

Altogether, our data unambiguously demonstrated that the majority of neoblasts in *M. lignano* distribute their DNA randomly during cell division, and that label-retention is a direct result of cellular quiescence, rather than a sign of co-segregation of labeled strands.

## Introduction

Adult stem cells (ASCs) have a long-term and dual responsibility to both self-renew and produce differentiated progeny, thereby playing a crucial role during the entire lifetime of an organism [1], [2]. Given the constant demand for proliferation and the error-prone nature of DNA replication, these cells possess a high risk for malignant transformation [3]. As a consequence, it has long been postulated that ASCs might have acquired specialized features to protect their genome [4], [5]. A highly efficient DNA-repair system is commonly described as a stem cell trait, which would serve this purpose [2].

**Figure 1:**
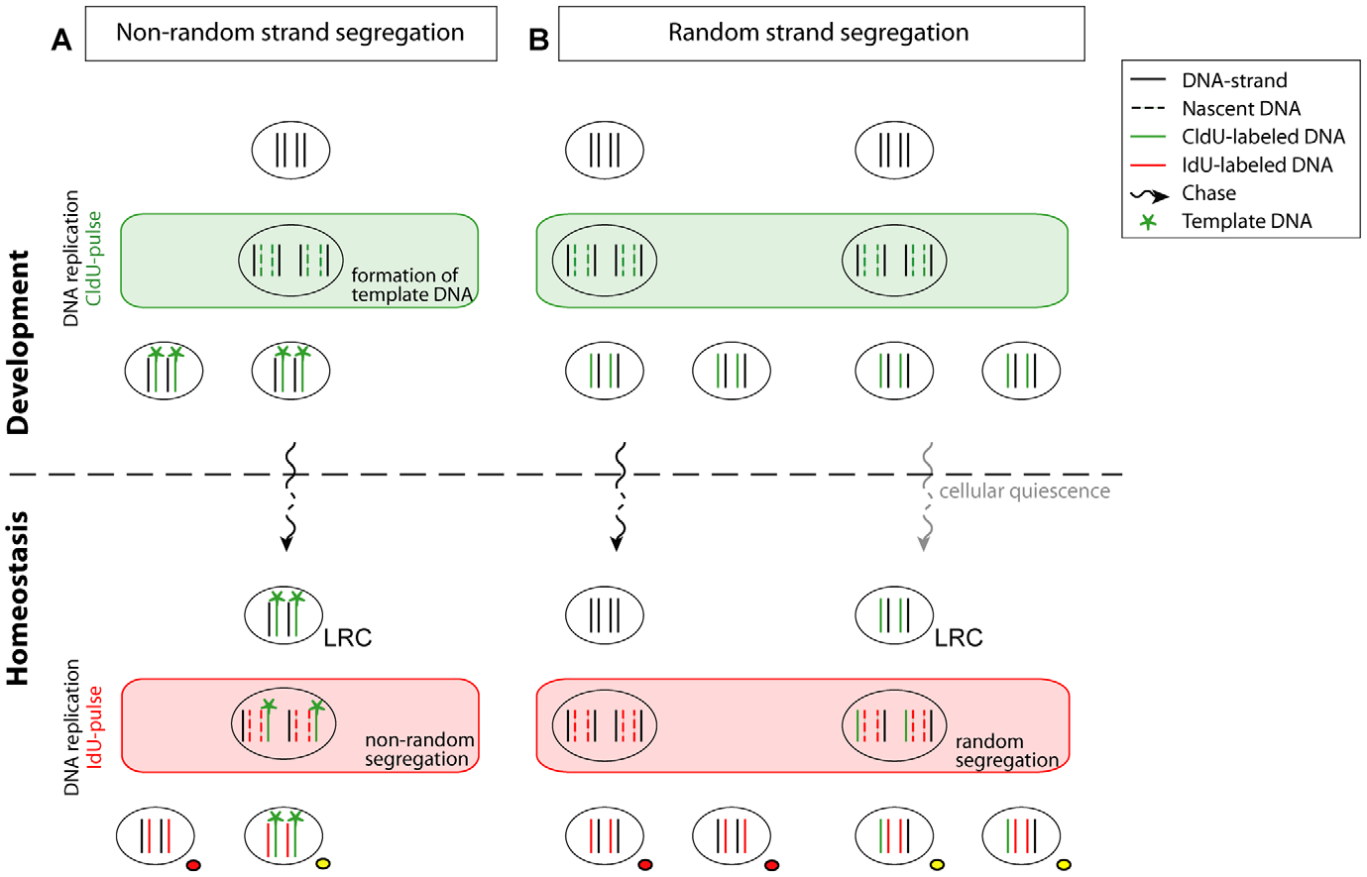
Possible interpretation of label-retention studies, using a double labeling approach. (A): Cairns' theory, known as the immortal strand hypothesis, postulates that adult stem cells (ASCs) segregate their DNA-strands non-randomly and permanently retain original template DNA. Using a thymidine derivate such as CldU, these template DNA strands can be labeled at the moment they are synthesized during development, which results in two daughter cells in which complementary DNA-strands are composed of 1 labeled template strand next to an unlabeled normal DNA strand. Because of co-segregation of the labeled template DNA-strands, from the second division after establishment of labeled immortal strands on, the label is passed on to only one daughter cell. Therefore, cells are able to retain label indefinitely during adulthood and are referred to as label-retaining cells (LRCs). By performing a second pulse with another thymidine analog such as IdU, LRCs can become double labeled. (B): If DNA is segregated randomly, labeled DNA-strands which are created during a first pulse period with CldU, are distributed over both daughter cells, instead of only one. By consequence, in a regularly cycling cell, the label is diluted under the detection threshold after a certain number of cell cycles in CldU-free medium (left panel). Thus, initially-labeled cells do not retain the label and a second pulse-period with IdU will not result in double labeled cells. However, if cells remain quiescent after they incorporated CldU, the chance of dilution of the label is reduced due to low or even absent cell proliferation (right panel). This results in LRCs. Creating double labeled LRCs after a second pulse with a thymidine derivate (IdU), is therefore possible, yet unlikely because of low cell cycle activity. Abbreviations: LRC, label-retaining cell; CldU, 5-chloro-2′-deoxyuridine; IdU, 5-iodo-2′-deoxyuridine.

Additionally, a putative mechanism by which ASCs might limit accumulating erroneous genetic information, was originally proposed by Cairns [6] as the immortal strand hypothesis. According to this hypothesis, stem cells segregate their DNA strands non-randomly upon asymmetric self-renewing cell divisions. Those sister chromatids containing the original template DNA strands are selectively retained in one daughter cell, destined to be the renewed stem cell. The newly synthesized strands, which might have acquired mutations during replication, are passed on to the tissue committed cell. A common strategy to verify this hypothesis, relies on pulse-chase studies with nucleotide tracers, such as tritiated thymidine, bromodeoxyuridine (BrdU), or chlorodeoxyuridine (CldU). Labeling the original ‘immortal’ DNA strands when they are synthesized during development or regeneration, should result in ‘Label-Retaining Cells’ (LRCs), considering that these labeled strands are co-segregated during cell divisions (Figure 1A, top panel).

Alternatively, retention of label in stem cells can likewise be explained as a result of cellular quiescence. Restricting the number of stem cell divisions seems an equally valuable mechanism for preservation of genome integrity and furthermore prevents stem cell exhaustion [7]–[12]. Low or absent proliferative activity, after cells were labeled with nucleotide tracers, reduces the chance of label-dilution and allows quiescent ASCs to be identified as ‘Label-Retaining Cells’ (LRCs) (Figure 1B, top right panel). Conversely, in more rapidly cycling progeny cells the label is gradually diluted (Figure 1B, top left panel). Performing a double labeling protocol using a second nucleotide tracer serves as a promising tool to assess information on the proliferative activity of LRCs (Figure 1A, B, bottom panel).

Elucidating the label-retention theory remains a matter of intense debate, fueled by publications confirming the theory of cellular quiescence on one hand [9], [13]–[17], versus those supporting non-random segregation of DNA strands on the other hand [18]–[23]. It has been shown that culture environments can alter the patterning of cells in ways that modify their fates and proliferative potential [24], [25]. Therefore, the use of model organisms in which stem cells can be studied *in vivo* has attracted substantial attention [26]–[31]. However, the *in vivo* data on this topic is mainly gathered in systems in which the analysis of stem cell behavior is hindered by the rare incidence of stem cells, relative inaccessibility of these cells for experimental manipulation *in vivo*, and lack of specific stem cell markers [32].

**Figure 2:**
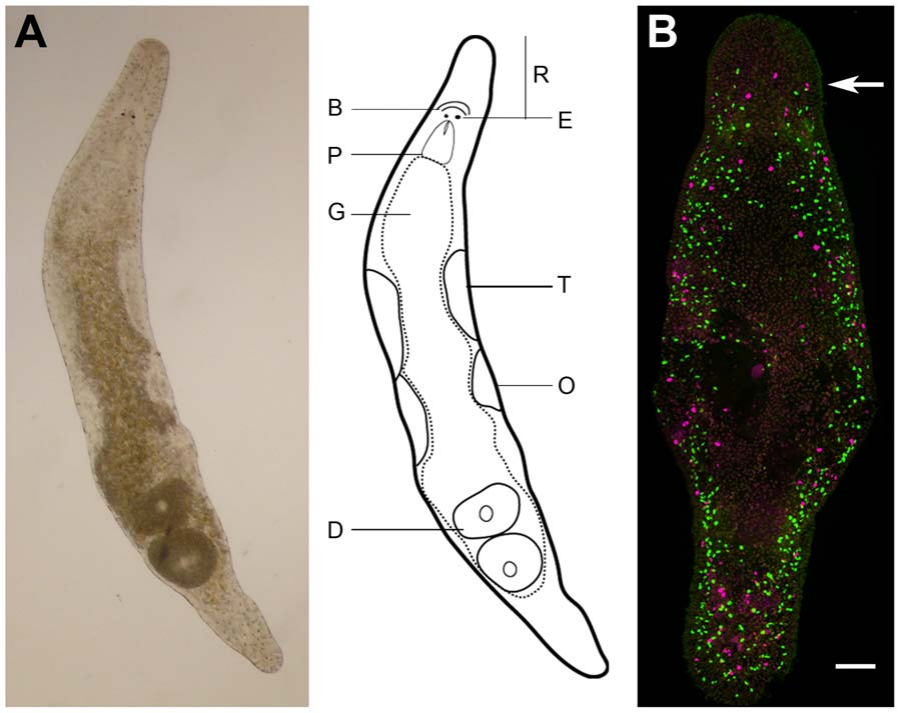
*Macrostomum lignano* (Platyhelminthes). (A): Light microscopic picture of an adult specimen, dorsal view (left panel). Schematic drawing (right panel). Abbreviations: R, rostrum; B, brain; E, eye; P, pharynx; G, gut; T, testis; O, ovary; D, developing egg. (B): Confocal projection of a double BrdU/phospho histone H3 immunostaining (green S-phase cells, red mitoses converted to magenta) after a 30-min BrdU pulse (no chase) in an adult animal. During homeostasis, proliferating neoblasts are distributed in a bilateral pattern. S-phase, nor mitotic cells are visible anterior to the eyes. Arrow indicates the level of the eyes. Anterior is on top. Scale bars: 50 µm.

Over the last decennia, flatworms have been put forward as valuable model organisms to unravel the complex biology of stem cells [33]–[37]. These simple, triploblastic metazoans exhibit a powerful stem cell system that is maintained through adult life and which lies at the root of their exceptional developmental plasticity and regeneration capacity [38]. The flatworm stem cell population is comprised of pluripotent stem cells, referred to as neoblasts, which remain mitotically active during adulthood, unlike all differentiated cells in the organism [39]–[44]. Among flatworms, *Macrostomum lignano* (Figure 2A) has been recently described as a highly advantageous model for *in vivo* stem cell research [37], [45]–[51]. Advantages are the ease of culturing [45], [51], the short embryonic and post-embryonic development (5 and 14 days, respectively), and the limited number of cells (25 000 in total) which facilitates cell quantification [37]. Furthermore, neoblasts are well characterized and present in large numbers (6.5% of the total cell number) [52]. They can easily be distinguished from non-stem cells, based on morphological traits, and by using using an antibody against neoblast-specific Macvasa proteins [45], [48]. Immunohistochemical staining of S-phase neoblasts with the thymidine analog bromodeoxyuridine (BrdU), and mitotic neoblasts with an anti-phospho histone H3 mitosis marker (anti-phos-H3), have revealed a bilateral distribution of these cells [45], [46] (Figure 2B). Pulse and pulse-chase studies with thymidine analogs such as BrdU can easily be performed by soaking the animals in the analog-containing medium during the pulse period. Moreover, an *in vivo* double labeling technique using two different thymidine derivates, iododeoxyuridine (IdU) and chlorodeoxyuridine (CldU), can be applied [37]. To our knowledge, this technique has been performed only once before to test the segregation mode of DNA-strands *in vivo* [53]. Altogether, these advantages enable *in vivo* analysis of the exact mode of DNA segregation in ASCs in the flatworm *M. lignano*.

We aimed to elucidate if label-retaining stem cells exist in *M. lignano*. Performing long-term pulse-chase studies, four different *in vivo* approaches were used. First, a single BrdU-pulse-chase experiment was performed to demonstrate the existence of LRCs. Second, among this population of LRCs, Macvasa-positive (Macvasa^+^) neoblasts were identified. Third, double labeling of the LRCs with chlorodeoxyuridine (CldU) and iododeoxyuridine (IdU) gave information on the proliferative activity. Finally, the actin inhibitor cytochalasin D was used to directly analyze the segregation of labeled DNA strands at the single-cell level. Altogether, our results demonstrate that in *M. lignano* random segregation of DNA strands is predominant, and that label-retention is a direct result of cellular quiescence.

## Methods

### Animal Culture

Cultures of *M. lignano* were reared in standard culture medium (f/2) [54] as described previously [51], [55]. To obtain animals of a standardized age, adult worms were put together for 24 hours, after which the eggs were collected. Animals that were pulsed with a thymidine analog, were protected from light.

### BrdU-pulse labeling and immunocytochemistry in whole-mount organisms and macerated cell suspensions

A 24-hour incubation period in the thymidine analog 5-bromo-2′-deoxyuridine (BrdU - Sigma) was given to 11 standardized age groups of embryos (at day 1, 2, 3, 4, or 5 of development) and hatchlings (at day 6, 7, 8, 9, 10, or 11 of development). Together, the initial five age groups cover the embryonic development of *M. lignano*, while the following groups cover the first six days of post-embryonic development. Both embryos and hatchlings were pulsed, simply by soaking them in f/2 containing BrdU (500 µM). Animals were then kept in in standard culture medium, in the presence of food (*ad libitum*), for two or six months in the absence of BrdU. Subsequently, BrdU positive cells were localized using the protocol described below.

The procedure for visualization of the incorporated BrdU in whole mount animals was modified from a previous publication [45]. Specimens were relaxed in MgCl_2_ (1∶1 MgCl_2_.6H_2_O (7.14%):f/2, 5 min – MgCl_2_.6H_2_O (7.14%), 5 min), fixed in 4% paraformaldehyde (PFA, 30 min), and rinsed in PBS-T (phosphate-buffered saline+0.1% Triton X-100, 3×10 min). Animals were then treated with Protease XIV (0.2 mg/ml in PBS-T, 37°C, under visual control) and DNA was denatured with 2 N HCl (1 h, 37°C). Rinsing with PBS-T (6×10 min) and blocking in BSA-T (PBS-T+1% bovine serum albumin, 30 min) were followed by overnight incubation in the primary antibody, rat-anti-BrdU (1∶800 in BSA-T, 4°C - AbD Serotec). Subsequently, animals were washed in PBS-T (3×10 min) and incubated in the secondary antibody, FITC-conjugated donkey-anti-rat (1∶600 in BSA-T, 1 h – Rockland). Finally animals were rinsed in PBS (3×10 min) and mounted in Vectashield (Vector Laboratories).

Simultaneous visualization of S- phase and mitotic neoblasts, using anti-BrdU and anti-phos-H3, was performed as described by Nimeth *et al.* [46], with the exceptions that both the Protease XIV and HCl treatment was performed as described above. Rhodamine-conjugated goat-anti-rabbit (1∶150 in BSA-T, 1 h – Millipore) was used as a secondary antibody for the mitosis marker.

In macerated cell suspensions, the incorporated BrdU was visualized as described before [45], though some modifications were made. Twenty animals were incubated in 100 µl of maceration solution (glacial acetic acid∶glycerol∶distilled water 1∶1∶13 - 9% sucrose, 10 min), after which calcium/magnesium-free medium (CMF, 100 µl) was added. Thirty minutes after addition, animals were gently pipetted until they fell apart into single cells. Cells were then pelleted (130× g, 20 min), supernatant was removed, and the pellet was resuspended in PBS (200 µl). The cell suspension was spread onto poly-L-lysine coated slides. The staining of BrdU, was performed directly on these slides in a humid chamber, and was identical to the protocol for whole-mount preparations, with the exclusion of the Protease XIV step. Prior to the mounting of slides with Vectashield, DNA was stained using DAPI (1 µg/ml in PBS, 1 h). The morphology of single cells was studied, following the methods described earlier [45], [56]. Neoblasts were identified as small, rounded cells (5–10 µm) with a large nucleus and scanty cytoplasm.

### Double labeling with CldU and IdU and immunocytochemistry of whole mounts

Standardized age groups of embryos and hatchlings were pulsed with the thymidine analog 5-chloro-2′-deoxyuridine (CldU; 500 µM in f/2, 24 h - Sigma) following the same protocol as described for BrdU-labeling. Animals were then chased for six months in the presence of food (*ad libitum*) in CldU-free standard culture medium, after which they were pulsed with 5-iodo-2′-deoxyuridine (IdU; 50 µM in f/2 – Sigma) continuously for 7 days. The following steps were identical to the single BrdU-labeling in whole-mount animals (starting from MgCl_2_-relaxation until incubation in BSA-T). Animals were then incubated in the first primary antibody, mouse-anti-IdU (1∶800 in BSA-T, overnight, 4°C - Becton-Dickinson); washed in PBS-T (3×10 min); incubated in stringency buffer (0.5 M NaCl+36 mM Tris HCl+0.5% Tween 20, 15 min) for removal of nonspecifically-bound primary antibody; and washed again in PBS-T (3×10 min). Subsequently, specimens were incubated in the first secondary antibody, Alexa Fluor 568-conjugated goat-anti-mouse (1∶900 in BSA-T, 1 h - Invitrogen); the second primary antibody, rat-anti-CldU (1∶800 in BSA-T, overnight, 4°C - AbD Serotec); and the second secondary antibody, FITC-conjugated donkey-anti-rat (1∶600 in BSA-T, 1 h - Rockland). Incubation-periods in antibodies were separated by washing steps in PBS-T (3×10 min). Finally, animals were rinsed in PBS (3×10 min) and mounted in Vectashield.

### EdU-pulse labeling, immunocytochemistry in macerated cell suspensions and the use of cytochalasin D

Embryos and hatchlings, standardized by age, were soaked in the thymidine analog 5-ethynyl-2′-deoxyuridine (EdU; 20 µM in f/2 - Invitrogen) during development, respectively from day 1 until day 5 continuously, and from day 6 until day 11 continuously. Animals were then chased in the presence of food (*ad libitum*) for two months in EdU-free medium, followed by a seven-day incubation period in the actin-binding protein cytochalasin D (5 µM in f/2 - Sigma). Subsequently, they were macerated, following the protocol described earlier and cells were spread onto poly-L-lysine coated slides, washed in PBS (3×10 min), blocked with BSA-T-1% (PBS+1% Triton X-100+1% BSA, overnight, 4°C) and incubated in Click-iT® EdU reaction cocktail (concentrations according to manufacturer's instructions - Invitrogen). Afterwards, slides were washed thoroughly in BSA-T-1% (1 h), DNA was stained with DAPI (1 µg/ml in PBS, 1 h) and cells were mounted using Vectashield (Vector Laboratories).

### EdU-pulse labeling, immunocytochemistry in whole mounts and the use of Macvasa antibody

An EdU-pulse was performed as described above, in embryos (day 1–5, continuously) and hatchlings (day 6–11, continuously), after which a three-month chase was performed in the presence of food in EdU-free medium. Subsequently, animals were relaxed, fixed, and rinsed with PBS-T, as described above. Blocking was performed with BSA-T (2 h), followed by incubation in Click-iT® EdU reaction cocktail (concentrations according to manufacturer's instructions – Invitrogen). Next, Macvasa^+^ cells were visualized as described by Pfister *et al.* [48], using primary rabbit-anti-Macvasa and secondary TRITC-conjugated goat-anti-rabbit. Finally, animals were rinsed in PBS (3×10 min) and mounted in Vectashield.

### Imaging and quantification of LRCs in whole-mounts

Epifluorescence and phase-contrast microscopy was performed on a Zeiss Axiovert 200 M inverted microscope, followed by image processing using AxioVision 4.7.2. software (Zeiss) and Photoshop CS2. A Nikon Eclipse C1si confocal microscope was used for generating confocal images of whole mount animals. An argon laser (488 nm) in combination with a narrow band-pass filter (BP 515/30), and a helium-neon laser (543 nm) in combination with a narrow band-pass filter (BP 593/40) were used for visualization of the FITC-fluorochromes (CldU) and the Alexa Fluor 568-fluorochromes (IdU), respectively. Images where processed, using Nikon EZ-C1 3.40 software and Adobe Photoshop CS2.

Quantification of BrdU^+^ LRCs was performed on confocal images, using the free software program Image J [57]. Images were prepared by performing automatic thresholding, after which cells were quantified automatically, using the ‘Analyze Particles’ plug-in in Image J. In order to exclude labeled differentiated cells from the cell counts, exclusion parameters were activated based on size and shape of the labeled particles (Size pixel ∧2: 10–100; Circularity: 0.80–1.00). Based on their location in regions which are known to lack neoblasts in *M. lignano* [45], labeled cells in the rostrum (anterior to the eyes) and at the median axis were not considered to be stem cells and were therefore excluded from counts.

Statistical analysis was performed using Mann-Whitney U (BrdU^+^ LRC's) and Kruskal-Wallis (CldU^+^/IdU^+^ LRC's) tests.

## Results

### Establishment of LRCs in *M. lignano*

**Figure 3:**
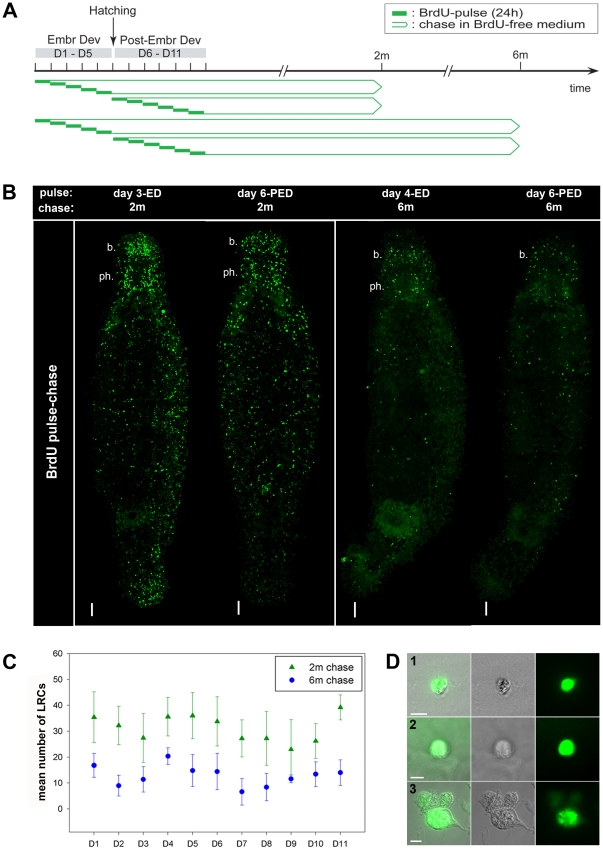
LRCs can be established during both embryonic and post-embryonic development, and lose their label at a slow rate. (A): Scheme of the experimental set-up. Animals were pulsed with BrdU (24 h) at successive 24-hour time frames of embryonic and post-embryonic development, followed by chase times of 2 and 6 months in BrdU-free medium. Subsequently BrdU was visualized and the presence of LRCs was analyzed. (B): Visualization of LRCs (green) in whole mount animals (confocal projections of BrdU immunostaining). Left panel, from left to right: animal pulsed at day 3 (ED), animal pulsed at day 6 (PED); both animals were chased for 2 months. Right panel, from left to right: animal pulsed at day 4 (ED), animal pulsed at day 6 (PED); both animals were chased for 6 months. Abbreviations: b., cluster of BrdU^+^ cells at the level of the brain; ph., cluster of BrdU^+^ cells at the mouth-pharynx region. Anterior is on top. (C): Graph representing the quantification of LRCs (mean number+Standard deviation, n = 22×5), chased for 2 and 6 months. LRCs were present in all animals of all pulse groups, both after two and six months. In animals, chased for 6 months, a significant lower number of LRCs was present, compared to animals that were pulsed for 2 months. Abbreviations: BrdU, 5-bromo-2′-deoxyuridine; ED, embryonic development; PED, post-embryonic development. (D): Visualization of LRCs in macerated cell suspensions. Superimposition of interference contrast and fluorescence images of BrdU immunostaining (left), interference contrast images (middle) and fluorescent images (right). Animals were pulsed during embryonic and post-embryonic development, and chased for 2 months. Pictures show labeled neoblasts with a large nucleus surrounded by a small rim of cytoplasm (C1, C2), and a labeled nerve cell (C3). Scale bars: B, 50 µm; D, 5 µm.

To evaluate whether LRCs were present in *M. lignano*, animals were pulsed with BrdU during development, allowing nascent neoblasts to incorporate the thymidine analog into their DNA. For BrdU-incorporation, five groups of embryos and six groups of hatchlings, standardized by age, were pulsed at successive 24-hour time intervals during both embryonic (days 1–5) and postembryonic (days 6–11) development (Figure 3A). This wide developmental window was chosen to ascertain that the potential founder label retaining neoblasts were covered by the pulse period. In order to pinpoint a specific time interval during which these neoblasts originate, BrdU-incubation was limited to intervals of 24 hours. Following the BrdU-pulse, specimens were chased in the presence of food, for two and six months in BrdU-free medium. Subsequently, after both chase periods, 10 randomly chosen animals of every pulse-group (days 1–11) were sacrificed, BrdU was visualized, and animals were examined for the presence of LRC's.

After two months of chase, the presence of cells that had retained the BrdU label was confirmed in all studied animals that were pulsed during embryonic and post-empbryonic development (total n = 110). LRCs were distributed all over the body (Figure 3B, left panel). A high density of BrdU^+^ cells was observed in a bilateral pattern, which is in accordance with the distribution of neoblasts in *M. lignano* (Figure 2B) [45]. Outside this bilateral pattern, two separate clusters of labeled cells were found; one at the level of the brain, and another at the level of the mouth and pharynx. To test whether LRCs could be established during homeostasis as well, adult individuals were pulsed with BrdU and then chased for two months. Similarly, this resulted in the presence of LRCs in all studied individuals (data not shown).

After a six-month chase-period, 95% of a total of 110 randomly chosen animals could be labeled (n = 104), and LRCs were present in all of them. These labeled cells were scattered throughout the body (Figure 3B, right panel). An accumulation of labeled cells, similar to those after two months chase, at the brain region, the mouth-pharynx region, or both was visible in 75% of the animals.

For every pulse-group, both at two and six months chase, the number of LRCs was quantified in five animals (Figure 3C), as described in the Methods section. The number of LRCs was significantly lower in all pulse-groups at six months chase when compared to the same groups after two months chase (for all pulse-groups, p<0.05). Thus, a considerable amount of LRCs have lost their label over time, meaning that these cells are not able to retain label indefinitely, or labeled cells were replaced by the progeny of unlabeled neoblasts during tissue homeostasis. The number of LRCs after two months chase seemed to vary, with a mean value of 31 LRCs per animal (±9, n = 55). After 6 months chase, the mean number of LRCs for all pulse groups combined was 13 per animal (±6, n = 55).

These LRCs might represent differentiated progeny of labeled stem cells, in which case the label is retained due to the post-mitotic state of differentiated cells in flatworms. In order to verify whether LRCs, or a fraction thereof, could be identified as neoblasts, two month chased animals were macerated into single cells and BrdU was visualized. By analyzing the morphology of BrdU-positive cells, the existence of label-retaining neoblasts was confirmed (Figure 3D_1,2_). In addition, several differentiated labeled cells were found, including cells displaying the morphology of nerve cells (Figure 3D_3_) and epidermis cells (not shown).

### Label-retaining stem cells are positive for the neoblast marker Macvasa

An additional experiment was performed to test whether neoblasts could be identified within the population of LRCs. For this purpose, a polyclonal antibody against a homolog of the highly conserved Vasa protein of *M. lignano* (Macvasa) was used. Unlike in other metazoans, where Vasa is almost exclusively detected in germ line cells, Macvasa in *M. lignano* is also present in a subset of somatic stem cells in a characteristic pattern - a ring of Macvasa-labeled spots of nuage surrounding the nucleus [48]. Consequently, Macvasa can be used as a neoblast marker in this flatworm. In this experiment, LRCs were established using EdU, since HCl-denaturation is unnecessary for the visualization of this thymidine analog, which enabled simultaneous labeling of Macvasa proteins. The specificity of EdU-labeling was observed comparable to BrdU (see Figure S1, Text S1).

**Figure 4:**
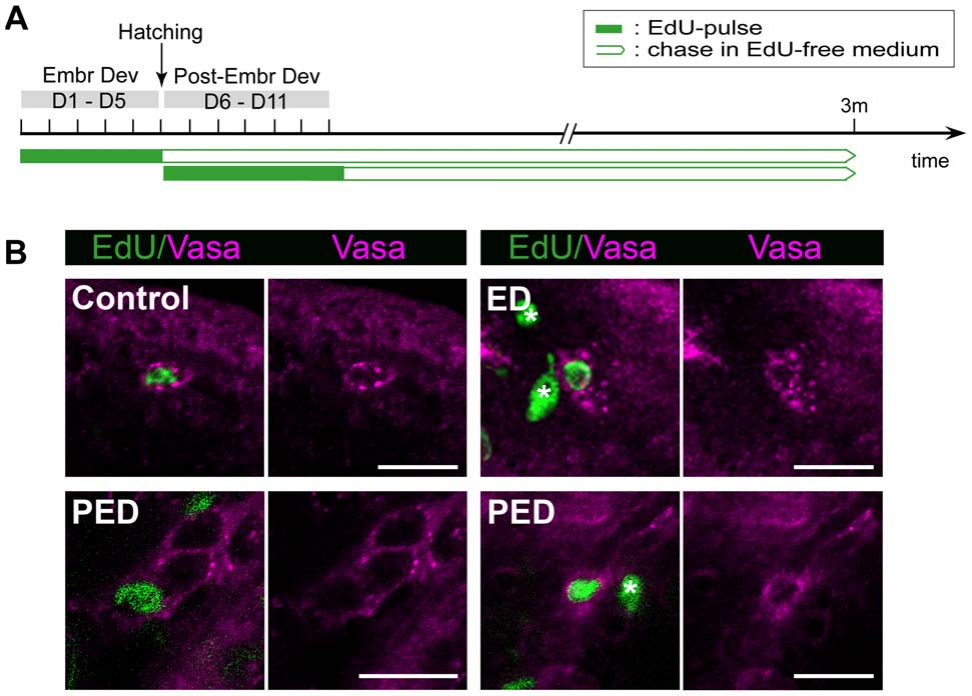
Identification of label-retaining neoblasts (Macvasa). Identification of neoblasts among the population of LRCs, using an antibody against neoblast-specific Macvasa proteins. (A): Scheme of the experimental set-up. Animals were pulsed continuously with EdU during embryonic development (day1–day5) and during post-embryonic development (day6–day11), followed by a chase time of 3 months in EdU-free medium. Subsequently, EdU was visualized in combination with Macvasa. (B): Visualization of label-retaining neoblasts in whole-mount animals (confocal image). LRCs (EdU) are green. The TRITC-signal of the Macvasa proteins, was converted to magenta. EdU^+^/Macvasa^+^ cell in a control animal (no chase) displays Macvasa proteins in a ring of nuage around the nucleus (upper left panel). EdU^+^/Macvasa^+^ cell in an animal pulsed during ED (upper right panel). EdU^+^/Macvasa^+^ cells in an animal pulsed during PED (lower left and right panel). LRCs that are Macvasa-negative (asterisks) are visible in individuals pulsed during ED and PED (right panels). Abbreviations: EdU, 5-ethynyl-2′-deoxyuridine; Vasa, Macvasa; ED, embryonic development; PED, post-embryonic development. Scale bars: 10 µm.

In the first pulse group, individuals were pulsed continuously with the thymidine analog EdU for five days during embryonic development (day 1 until day 5). A second group of individuals was pulsed continuously with EdU during the first six days of post-embryonic development (day 6 until day 11). Individuals were then chased for three months, in the presence of food (Figure 4A), after which EdU-positive (EdU^+^) and Macvasa^+^ cells were visualized.

Macvasa^+^ LRCs were identified in all individuals pulsed during embryonic (day 1–day 5) as well as in individuals pulsed during post-embryonic development (day 6–day 11) (Figure 4B). Macvasa protein in double labeled cells, was visible in spots of nuage around the EdU-labeled nucleus, as observed previously [48].

Macvasa^+^ LRCs were located at the lateral sides of the animal, the area described to contain somatic neoblasts [45]. Double positive cells were never observed in the testes, nor ovaries.

In conclusion, these results directly confirm the existence of neoblasts among the population of LRCs, which are distributed among other somatic neoblasts.

### Label-retaining stem cells manifest low proliferative activity

**Figure 5:**
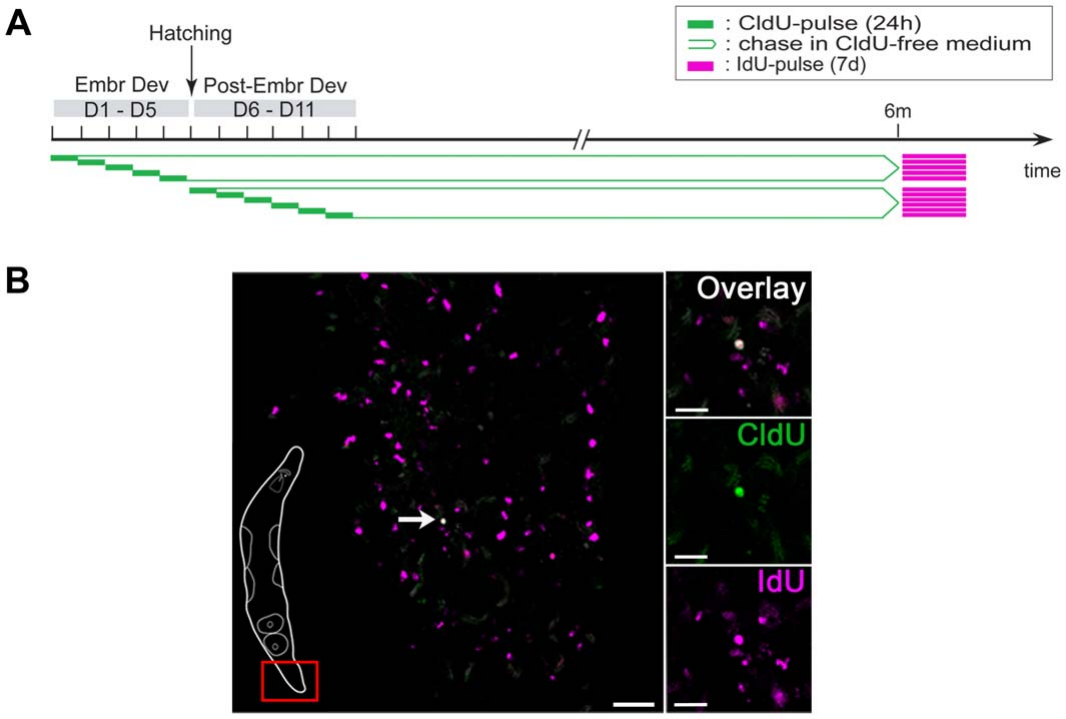
Low proliferative activity of label-retaining cells (LRCs). Analysis of the proliferative activity of LRCs in *M. lignano*, performing a double labeling technique with the proliferation markers CldU and IdU. (A): Scheme of the experimental set-up. Animals were pulsed with CldU (24 h) at successive 24-hour time frames during embryonic and post-embryonic development, followed by a chase time of six months in CldU-free medium. Subsequently animals were pulsed for 7 days continuously with IdU, after which both markers were visualized and the presence of double labeled cells was analyzed. (B): Double labeled LRC located in the tail region (left inset), in a whole mount animal (confocal plane) that was pulsed with CldU (green) on day 1 of embryonic development, chased for six months, and pulsed again with IdU (red, converted to magenta). Every LRC (labeled during the first pulse) that proliferates during the second pulse will become overlabeled with IdU, and is CldU^+^/IdU^+^ (white, indicated with arrow). LRCs that do not proliferate during the second pulse are CldU^+^ (green) and cells which are proliferating during the second, but not the first pulse, are IdU^+^ (magenta). Abbreviations: CldU, 5-chloro-2′-deoxyuridine; IdU, 5-Iodo-2′-deoxyuridine. Scale bars: C, 50 µm; C inset, 20 µm.

To further analyze the proliferative activity of label-retaining stem cells, a CldU/IdU double labeling method was applied to label the S-phase of stem cells after a six months chase time. In these experiments, CldU was administered continuously for 24 hours to different groups of embryos and hatchlings at successive time frames of embryonic (days 1–5) and post-embryonic (days 6–11) development, followed by a chase time of six months in CldU-free culture medium. Following the chase period, a second pulse with IdU was performed for seven days continuously to embrace all LRCs that proliferated during this week (Figure 5A). Immediately afterwards, animals were immunostained for CldU and IdU. Consequently, every CldU^+^ LRC going through S-phase during the second pulse period with IdU incorporates this thymidine analog as well and therefore becomes double labeled.

The presence of proliferating LRCs (CldU^+^/IdU^+^ cells) was confirmed in representatives of every pulse group (day 1–11) (Figure 5B). Since neoblasts are the only somatic cells that are actively dividing in *M. lignano*, this directly proves that each 24-hour pulse period resulted in neoblasts that were able to retain their label for six months. Hence, no specific time-frame could be pinpointed for the establishment of proliferating label-retaining stem cells. The distribution of all CldU^+^/IdU^+^ cells was in accordance with the normal distribution of neoblasts [45], except for two cells that were located in the rostrum. These two cells were probably differentiated and migrated during the seven day administration of the second pulse.

A quantitative study of double labeled cells was performed in animals that were pulsed during embryonic development (days 1–5) and chased for six months. Overall, the number of CldU^+^/IdU^+^ cells was very low, with an observed maximum of three double labeled cells per worm (8% of all animals observed, n = 38). In most animals (58%) zero double labeled cells were quantified, and 24% and 11% of all observed animals had one and two CldU^+^/IdU^+^ cells, respectively. When analyzed for each of the five pulse groups, the mean numbers of double positive cells per worm did not significantly differ between the groups (p>0.8). This low number of double labeled cells indicated little proliferative activity among the label-retaining stem cells.

### The use of cytochalasin D indicates random segregation of DNA strands

In order to directly test the segregation pattern of DNA strands *in vivo*, cytochalasin D was used. This actin binding protein blocks cytokinesis, while karyokinesis is unaffected, thereby maintaining one cell with two daughter nuclei.

**Figure 6:**
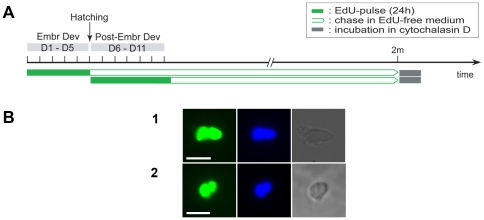
Random distribution of labeled DNA-strands among daughter nuclei of LRCs. Analysis of the distribution of labeled DNA-strands among daughter nuclei of label-retaining cells (LRCs) on single cell level, using the actin-binding protein cytochalasin D. Cytochalasin D is an actin-binding protein that inhibits cytokinesis, while karyokinesis remains unaffected. Thereby, binucleate cells are created, which enables analysis of the distribution of DNA-strands among daughter nuclei on single cell level. (A): Scheme of the experimental set-up. Animals were pulsed continuously with EdU during embryonic development (day1–day5) and during post-embryonic development (day6–day11), followed by a chase time of 2 months in EdU-free medium. Subsequently animals were soaked in cytochalasin D for 7 days, EdU was visualized and DNA was stained with DAPI. (B): Visualization of binucleate LRCs in macerated cell suspensions. Fluorescence images of EdU (left), DAPI (middle), and interference contrast images (right) of binucleate LRCs, pulsed during embryonic (B1) and post-embryonic (B2, B3) development. Binucleate EdU^+^ cells display equivalent EdU-signal in both daughter nuclei. Abbreviations: EdU, 5-ethynyl-2′-deoxyuridine. Scale bars: 5 µm.

In order to incorporate EdU in all cells, embryos were pulsed continuously during the whole embryogenesis (day 1 until day 5). In a second pulse-group, hatchlings were continuously treated with EdU from day 6 until day 11. Animals were then chased in the presence of food for two months and subsequently incubated in cytochalasin D for one week (Figure 6A). Immediately afterwards, animals were macerated into single cells and stained for EdU. As a consequence, each label-retaining stem cell that proliferated during this one week incubation-period was blocked, resulting in a binucleate cell. This made it possible to analyze the distribution of labeled strands in the daughter nuclei.

All EdU^+^ binucleate cells that were observed displayed an equivalent EdU-signal in both daughter nuclei (Figure 6B). No cells were found that contained a labeled nucleus next to an unlabeled one, or otherwise displayed evidence of unequal fluorescence distribution.

## Discussion

In *M. lignano* four different *in vivo* approaches were used to analyze the exact segregation mode of DNA-strands during stem cell division. None of these approaches produced evidence for non-random segregation of DNA-strands, and were therefore inconsistent with the immortal strand hypothesis. In contrast, our long-term label-retention analyses are rather a confirmation of the existence of a population of relatively quiescent stem cells.

BrdU pulse-chase experiments were performed to test whether LRCs can be established in *M. lignano*. In order to enable pinpointing the origin of LRCs to a specific developmental window, an elaborate pulse scheme was designed. The data demonstrated that LRCs could be established in all specimen pulsed during 11 different time periods of development. A similar pulse-chase experiment in adult worms also resulted in LRCs. Thus, our study demonstrates that LRCs can be established in *M. lignano*, not only during the complete duration of embryonic development (day1–day 5), but also during post-embryonic development (day 6–day 11) and even during adulthood. Of possible concern was that label retention is caused by an artifactual withdrawal from the cell cycle, caused by a possible deleterious effect of the incorporated thymidine analog. However, our double labeling experiment contradicts this hypothesis, since label-retaining neoblasts are observed to proliferate. Previously, a continuous BrdU pulse (50 µM) from hatching to maturity in *M. lignano*, has been observed to result in viable labeled sperm [58]. Furthermore, other reports on the use of BrdU in *M. lignano* (with continuous pulse durations up to 14 d) have demonstrated no major effect on the dynamics of proliferating cells, since pulsing was not observed to affect morphology, animal behavior, cell cycle dynamics of fast cycling cells, differentiation of BrdU^+^ cells, and sperm production and differentiation [45], [46], [52], [58], [59]. Based on the results presented in this study and in previous studies, we can conclude that the effect of analog incorporation is minimal and that label-retention is not caused by a cell cycle arrest. Another possible caveat of label-retention studies is that the label is retained due to the post-mitotic state of differentiated cells. Working with *M. lignano*, however, enables identification of stem cells, based on their morphology [45], expression of *Macvasa* [47], and their ability to incorporate a thymidine analog, as the only proliferating somatic cells [45]. In this study, the presence of label-retaining neoblasts among the LRCs was proven in three ways: (1) labeled neoblasts were identified morphologically, (2) Macvasa proteins were demonstrated in a subset of LRCs, and (3) a small number of LRCs were observed to incorporate IdU in our double labeling experiment. These double labeled cells were found in all pulse groups, meaning that every pulse that was performed resulted in stem cells which retained label for extended periods of time. Thus, if label-retention would be a result of non-random segregation of labeled DNA-strands and ‘immortal’ strands do exist in *M. lignano*, our observations indicate that they would be synthesized continuously during embryonic and post-embryonic development, as well as during homeostasis. Serially creating new ‘immortal’ strands is totally incompatible with the purpose of the immortal strand hypothesis. On the other hand, the results of our analysis for the establishment of LRCs are compatible with the existence of a population of relatively quiescent stem cells. The inability to pinpoint the origin of LRCs to a specific developmental window, has previously been observed to be compatible with the existence of quiescent stem cell in mice [17].

**Figure 7:**
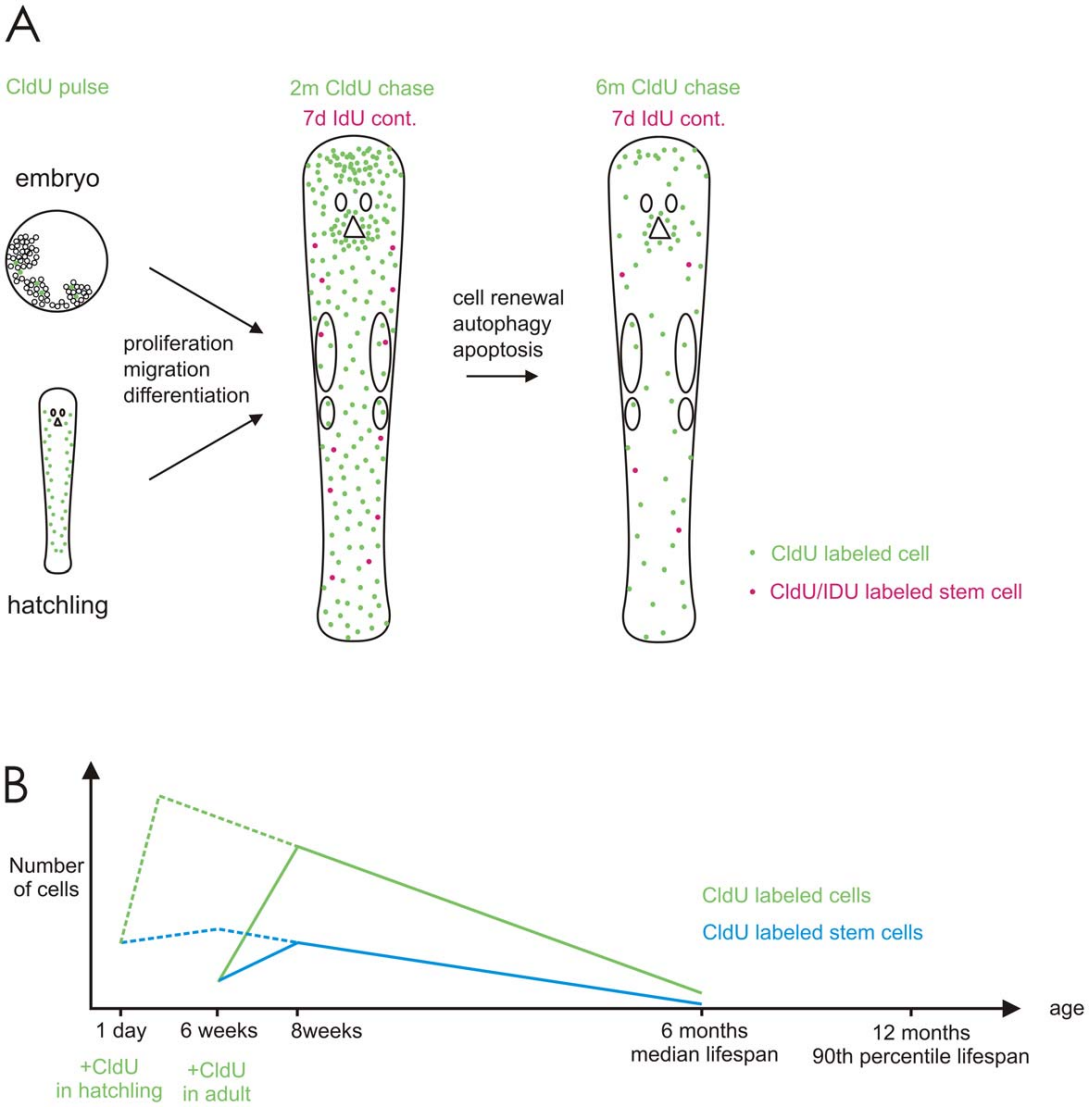
LRCs in M. lignano: their establishment, persistence and disappearance. A): Explanatory scheme of the results of a double labeling technique, using CldU and IdU. See text for details. (B): Explanatory graph representing the curve of the number of LRCs during the lifespan of *M. lignano*. Two starting points correspond to hypothetical pulses, one in hatchlings and one in adults. After performing a single pulse in hatchlings (dotted line curve) or adults (full line curve), a certain number of cells incorporate the thymidine analog and become labeled. Due to proliferation and differentiation this initial population of labeled cells expands. Their progeny either retains neoblast identity (blue) or loses mitotic activity to eventually become differentiated cells (green). After an initial period of expansion, the number of CldU-labeled cells decreases as a result of cell replacement from unlabeled progeny (green curve) and dilution of the CldU in proliferating neoblasts (blue curve). After six months, a small proportion of differentiated cells and neoblasts have retained the CldU-label, respectively due to long-term functionality and cellular quiescence.

Quantitative analysis of LRCs after two and six months chase demonstrates a significant decline in the number of LRCs. Still, neoblasts are observed to be able to retain label for as long as six months, a period equivalent to the median life span of *M. lignano* [60]. Thus, the label of LRCs is lost at an extremely slow rate, indicating little cell proliferation, a sign for cellular quiescence. To directly test the proliferative activity of LRCs during homeostasis, *in vivo* CldU/IdU double labeling experiments were performed. The extremely low numbers of double labeled LRCs demonstrate little proliferation. Both our single and double labeling experiment, therefore, deliver strong arguments for the existence of a population of quiescent stem cells in *M. lignano*. The combined outcome of our single and double labeling experiment after prolonged chase times, and the implications thereof, are explained in Fig. 7A. These experiments have demonstrated the establishment of a population of LRCs, consisting of labeled neoblasts on one hand, and differentiated progeny of labeled neoblasts on the other hand. By pulsing with CldU during embryonic or post-embryonic development, cells become labeled in S-phase (Fig. 7A, left panel). During successive development and growth, these CldU-labeled cells proliferate and create labeled progeny. The labeled progeny then migrates and differentiates to participate in homeostasis. As a result of proliferation, migration and differentiation CldU-labeled cells are distributed throughout the whole animal with some clustering at the brain and pharynx (Fig. 7A, middle panel). CldU labeled stem cells that go through S-phase during a second, 7 d-pulse period with IdU incorporate the second label. Due to cell renewal, autophagy and apoptosis, the amount of differentiated cells, and the amount of neoblasts that have retained label decreases with increasing chase time duration (Fig. 7A, right panel). In summary, this mode of cell turn over leads to the conclusion that random segregation of DNA-strands is the preliminary mechanism during neoblast divisions. Based on the results obtained from our label-retention study, a hypothetical graph is presented elucicating the persistance of labeled cells during the life span of *M. lignano* (Fig. 7B).

Finally, the actin-binding protein cytochalasin D was used to inhibit cytokinesis, thereby allowing analysis of the actual distribution of labeled DNA-strands among daughter cells of LRCs. This technique was performed for the first time *in vivo*. All binucleate cells observed, demonstrated equal distribution of labeled DNA-strands among daughter nuclei, indicating random segregation of DNA-strands in LRCs. However, it should be noted that if non-random segregation does occur, unequal distribution of fluorescence would not be visible until the second cell division after pulsing. Nonetheless, not one binucleate cell was found displaying non-random segregation of DNA-strands. Given the long chase time, and the fact that the number of LRCs was observed to decline with increasing chase time, it is unlikely that all binucleate cells divided only once after they were labeled during embryonic or post-embryonic development. In conclusion, this experiment corroborated the random distribution of DNA-strands in LRCs. The presence of a small population of quiescent neoblasts has been demonstrated previously in *M. lignano*. However, to date evidence was only produced for a short quiescent period of one week [52]. In irradiation studies on *M. lignano*, quiescent neoblasts that were activated upon radiation, were suggested to be responsible for recovery of the animals [61]. Our results, though, clearly demonstrate cellular quiescence on a considerably larger scale, since the foundation of a population of quiescent neoblasts appears to be already laid during the earliest stages of development. Moreover, these stem cells are shown to remain in this relatively quiescent state for a period as long as the median life-span in *M. lignano*.

In literature, adult stem cells are often suggested to exit the cell cycle upon reaching adulthood and form a dormant population of reserve cells. Additionally, developmental quiescence has been observed in a number of organisms. For example, in mice, the presence of quiescent or slow-cycling stem cells during the later stages of development has been observed in multiple tissues [62], [63]. It is not clear however, whether these cells remain in this dormant state during adulthood. Similarly, in lower organisms, cell cycle arrest has been described for vulval precursor cells during development in *C. elegans*. Still, these cells already resume proliferation during a later stage of development [64]. Thus, our observation of such an early onset of stem cell quiescence that persists for such a long time during adulthood, sheds light on a remarkable feature of neoblasts in *M. lignano* and opens venues for additional research.

Our study demonstrates that the neoblasts in *M. lignano* can be divided in at least two distinct subpopulations. The coexistence of quiescent and active neoblasts can serve to accomplish the two defining tasks of stem cell compartments, respectively maintaining a reserve for long-term repopulation, and creating progeny to cope with the high demand for proliferation. To date, it is not known whether these two populations are divided even further into a hierarchy of neoblast subpopulations with gradual limited differentiation potential. Such an organization of the stem cell pool has been postulated to greatly decrease the maximum number of cell divisions stem cells must undergo [2], hence reducing the risk of accumulating genomic errors. Furthermore, based on the high tolerance against radiation, neoblasts in *M. lignano* have been suggested to exhibit a highly efficient DNA repair system [61]. In its natural environment *M. lignano* is exposed to harsh environmental conditions such as e. g desiccation, very high or low salinity and temperatures. These stress conditions can damage DNA integrity. Therefore, *M. lignano* might have evolved competent DNA repair mechanisms that are indirectly highly beneficial for the stem cell system.

As previously mentioned, the immortal strand hypothesis is almost impossible to reject [32], [65]. Although this study produced evidence for quiescent stem cells and failed to detect non-random segregation of DNA-strands, it cannot be ruled out that only a proportion of the chromosomes are unequally distributed among daughter cells, as was reported by Armakolas and Klar [66]. In the same way, it can never be excluded that some rare cells in the neoblasts population display non-random segregation of DNA-strands. However, the biological relevance of such a system can be questioned if it is only present in a very limited number of cells.

In this long-term *in vivo* study, the exact mode of DNA-segregation during stem cell division was tested in the flatworm *M. lignano*. Altogether, our data suggest random segregation of DNA-strands and that label-retention is a direct result of cellular quiescence. We therefore conclude that the *M. lignano* stem cell system is protected by the presence of a population of quiescent neoblasts, probably together with a high capacity of DNA repair. Our findings contribute to a better understanding of how stem cell systems are organized in flatworms and higher organisms, including humans.

## Supporting Information

Figure S1 **BrdU/EdU double labeling.** To test the specificity by which EdU labels cells in S-phase, an EdU/BrdU double labeling was performed. Due to unequal binding kinetics of both analogs, however, a simultaneous pulse could not be performed. Instead, hatchlings and adults were pulsed with EdU (40 min), immediately followed by a pulse with BrdU (40 min). Subsequently both EdU and BrdU were visualized. (A, B): Visualization of EdU (green) and BrdU (red) in whole mount animals (epifluorescence). Labeled cells are EdU^+^/BrdU^+^, with the exception of a small number of EdU^+^/BrdU^−^ cells (arrowhead) and EdU^−^/BrdU^+^ cells (open arrowhead). These single labeled cells most likely represent cells that have left S-phase during the first pulse, and cells that have entered S-phase during the second pulse. (A): hatchling, complete animal. (B): adult, area of the gut. Abbreviations: EdU, 5-ethynyl-2′-deoxyuridine; BrdU, 5-bromo-2′-deoxyuridine. Scale Bars: 20 µm.
(TIF)

Text S1 **BrdU/EdU double labeling.** Supplementary method for BrdU/EdU double labeling.
(DOC)
