# Supplementary figures and images for: Stem Cells Propagate Their DNA by Random Segregation in the Flatworm Macrostomum lignano
Source: PLoS One. 2012 Jan 19;7(1):e30227. doi: 10.1371/journal.pone.0030227 (PMC3261893; doi:10.1371/journal.pone.0030227)

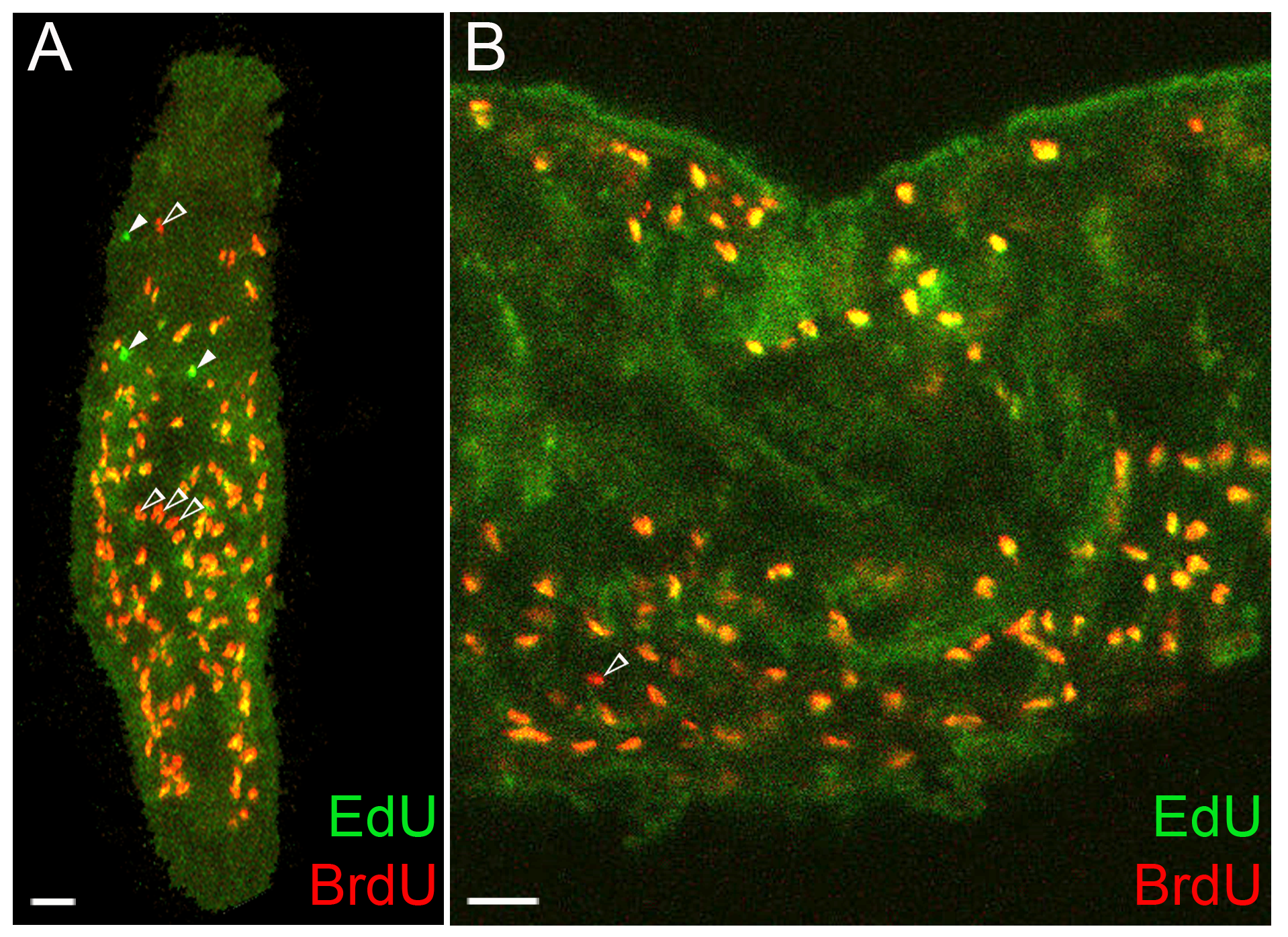

Supplement: Figure S1 — BrdU/EdU double labeling. To test the specificity by which EdU labels cells in S-phase, an EdU/BrdU double labeling was performed. Due to unequal binding kinetics of both analogs, however, a simultaneous pulse could not be performed. Instead, hatchlings and adults were pulsed with EdU (40 min), immediately followed by a pulse with BrdU (40 min). Subsequently both EdU and BrdU were visualized. (A, B): Visualization of EdU (green) and BrdU (red) in whole mount animals (epifluorescence). Labeled cells are EdU+/BrdU+, with the exception of a small number of EdU+/BrdU− cells (arrowhead) and EdU−/BrdU+ cells (open arrowhead). These single labeled cells most likely represent cells that have left S-phase during the first pulse, and cells that have entered S-phase during the second pulse. (A): hatchling, complete animal. (B): adult, area of the gut. Abbreviations: EdU, 5-ethynyl-2′-deoxyuridine; BrdU, 5-bromo-2′-deoxyuridine. Scale Bars: 20 µm. (TIF) [file pone.0030227.s001.tif]
