# Supplementary material for: Stem Cells Propagate Their DNA by Random Segregation in the Flatworm Macrostomum lignano
Source: PLoS One. 2012 Jan 19;7(1):e30227. doi: 10.1371/journal.pone.0030227 (PMC3261893; doi:10.1371/journal.pone.0030227)
Supplement: Text S1 — BrdU/EdU double labeling. Supplementary method for BrdU/EdU double labeling. (DOC) [file pone.0030227.s002.doc]

# Supplementary Text1

## Double labeling with EdU and BrdU

Animals were successively pulsed with EdU (40 min - Invitrogen) and BrdU (40 min – Sigma). Subsequently, animals were relaxed for 15 min with 7.14% MgCl_2_ and then fixed in paraformaldehyde (4% in PBS, 30 min). Next, specimens were washed with PBS-T, treated with Protease XIV and incubated in 2N HCl, as described for detection of BrdU. EdU detection with the Click-iT EdU Alexa Fluor (Invitrogen) was performed according to the manufacturer´s instructions. After washings steps PBS-T (), specimen were incubated with monoclonal anti-BrdU-antibody (1:1000 in BSA-T, overnight, 4°C - Roche). After washing with PBS-T (3 x 10 min), specimens were incubated in TRITC-conjugated swine-anti-rabbit (1:250 in BSA-T, 1 h, at room temperature - DAKO). Finally, specimen were washed with PBS-T (3 x 10 min) and mounted in Vectashield mounting medium (Vector Laboratories).
